# Good Cop, Bad Cop: The Different Roles of SRPKs

**DOI:** 10.3389/fgene.2022.902718

**Published:** 2022-06-02

**Authors:** Eleni Nikolakaki, Ioanna Sigala, Thomas Giannakouros

**Affiliations:** Laboratory of Biochemistry, Department of Chemistry, Aristotle University, Thessaloniki, Greece

**Keywords:** SRPK1, SRPK2, tumor suppressor, tumor promoter, chemotherapy sensitivity, chemotherapy resistance

## Abstract

SR Protein Kinases (SRPKs), discovered approximately 30 years ago, are widely known as splice factor kinases due to their decisive involvement in the regulation of various steps of mRNA splicing. However, they were also shown to regulate diverse cellular activities by phosphorylation of serine residues residing in serine-arginine/arginine-serine dipeptide motifs. Over the last decade, SRPK1 has been reported as both tumor suppressor and promoter, depending on the cellular context and has been implicated in both chemotherapy sensitivity and resistance. Moreover, SRPK2 has been reported to exhibit contradictory functions in different cell contexts promoting either apoptosis or tumor growth. The aim of the current review is to broaden and deepen our understanding of the SRPK function focusing on the subcellular localization of the kinases. There is ample evidence that the balance between cytoplasmic and nuclear SRPK levels is tightly regulated and determines cell response to external signals. Specific cell states coupled to kinase levels, spatial specific interactions with substrates but also changes in the extent of phosphorylation that allow SRPKs to exhibit a rheostat-like control on their substrates, could decide the proliferative or antiproliferative role of SRPKs.

## Introduction

There are more than 170 review articles in PubMed on signaling proteins that can function both as oncogenes or tumor suppressors in human cancer. The pro- and anti-tumorigenic activities depend on the cellular context and are usually ascribed on the different expression levels of these proteins and/or their involvement in different signaling pathways. Among these molecules are members of the CMGC family of protein kinases that share the common property to phosphorylate SR proteins and modulate their activity, thus regulating alternative splicing. DYRK2 has been reported to trigger antitumor and pro-apoptotic signals, while in parallel other studies identified DYRK2 as a highly overexpressed kinase in various cancer types and DYRK2 inhibitors were shown to exhibit antiproliferative properties (Tandon et al., 2021). This puzzling dual function of DYRK2 was mostly attributed to the phosphorylation of distinct substrates promoting either progression or suppression of tumors (Tandon et al., 2021).

SRPKs have also been related both to chemotherapy sensitivity and resistance (see analysis below). Moreover, SRPK1, the most-studied member of SRPKs, has been reported to function as both an oncogene and tumor suppressor depending on its expression level (Wang et al., 2014). Wang et al. (2014) proposed that aberrant SRPK1 expression in either direction induced constitutive Akt activation, thus implying that SRPKs can mediate tumorigenesis independently of their splicing effects, by modulating signaling pathways such as Akt. Yet, while SRPK1 has been reported to intervene in numerous signaling pathways in various cancers (Bullock and Oltean, 2017; Czubaty and Piekiełko-Witkowska, 2017; Nikas et al., 2019) none of these pathways has been implicated in the tumor suppressor function of the kinase nor was correlated with drug responsiveness.

The total SRPKs’ protein abundance in a cell has been considered as the determinant factor in their function. Yet, SRPKs are mobile proteins that can rapidly transit between the cytoplasm and nucleus. Their high response sensitivity to external signals, such as for example stress signals, is best evidenced by the fact that inefficient fixation with low concentrations of formaldehyde during the immunofluorescence process for just 5 min was suffient to stimulate nuclear translocation of SRPK1 in HeLa cells (Sigala et al., 2021a). Hence, the balance between nuclear and cytoplasmic pools of the kinases in a given cellular context is critical in determining cell behaviour. This fine-tuned compartmentalization is not only required to achieve phosphorylation of different target proteins but also to regulate the extent of phosphorylation of specific substrates. Given that SRPKs phosphorylate multiple serine residues within RS domains their mode of action is more akin to a rheostat than a binary switch, thus transducing changes in phosphorylation state into changes in protein function.

Here, we review the signaling pathways and the respective post-translational modifications that mediate the cytoplasmic-to-nuclear relocalization of SRPKs and discuss how these molecular mechanisms result in a well-defined change in nuclear kinase levels and activity which in turn may elicit specific functional outcomes. We also re-examine carefully the literature to shed some light onto the question why SRPKs have been reported both to promote and suppress cell proliferation and also to provide an explanation on the enigmatic and rather contradictory role of the kinases in mediating either sensitivity or resistance of cancer cells to chemotherapeutic drugs.

## SRPKs Localize Mainly in the Cytoplasm in Yeast and Cancer Cells

Our current knowledge regarding the subcellular localization of SRPKs is largely based on experiments in highly proliferating cells such as cancer and yeast cells. In cancer cells SRPK1 localizes predominantly in the cytoplasm with a more or less faint staining in the nucleus, while the nuclear staining profile of SRPK2 is more apparent (see for example Ding et al., 2006; Zhong et al., 2009; Sigala et al., 2021a). A similar cytoplasmic localization of Sky1 and dsk1 has also been observed in budding and fission yeast, respectively (Takeuchi and Yanagida, 1993; Siebel et al., 1999). The large non-conserved spacer region that separates the two catalytic kinase domains is required for localization of SRPKs in the cytoplasm during interphase, both in yeast and mammalian cancer cells, since its deletion results in almost exclusive nuclear localization of the mutant kinases (Takeuchi and Yanagida, 1993; Siebel et al., 1999; Ding et al., 2006). The spacer domains in SRPK1 and SRPK2, despite being quite variable in sequence, seem to function interchangeably in restricting the kinases in the cytoplasm (Ding et al., 2006). Mechanistically, the spacer region was shown to associate with molecular chaperones, thus anchoring the kinases to the cytoplasm (Zhong et al., 2009; Zhou et al., 2012). There are rather contradictory data regarding the anchorage mechanism between chaperones and SRPKs. Zhou et al. (2012) reported that in HeLa cells the Hsp70-containing complexes were responsible for anchoring and thus restricting the kinases in the cytoplasm, whereas the Hsp90-containing complexes facilitated SRPK translocation to the nucleus. Accordingly, Hsp90 knockdown prevented SRPK1 nuclear translocation. However, the same group reported earlier that inhibition of the Hsp90 ATPase activity induced dissociation of SRPK1 from the chaperone complexes resulting in translocation of the kinase from the cytoplasm to the nucleus (Zhong et al., 2009). In line with this report, Lu et al. (2015) have shown that, in a human liver carcinoma cell line, Hsp90 was responsible for anchoring SRPK2 in the cytoplasm, while Hsp90 knockdown or pharmacological inhibition promoted SRPK2 nuclear translocation.

The predominant cytoplasmic localization of SRPK1 and SRPK2 strongly supports the notion that these kinases mainly function outside the nucleus (Ding et al., 2006; Bustos et al., 2020). According to the classical view, cytoplasmic SRPKs phosphorylate newly synthesized SR proteins and facilitate their nuclear import *via* a specific member of the importin-beta family, transportin-SR2 (Lai et al., 2000; Lai et al., 2001). Besides SR proteins, RS or RS-like domains are also present in a variety of cytoplasmic proteins (Calarco et al., 2009; Bustos et al., 2020), including ZO-2 and RNF12 that have already been identified as SRPK1 targets (Quiros et al., 2013; Bustos et al., 2020). The nuclear fraction of SRPKs, in collaboration with CLKs, adjusts the degree of phosphorylation of SR proteins and thus modulates the splicing pattern of several genes. (Stamm, 2008; Aubol et al., 2016; Naro et al., 2021; Pastor et al., 2021). Furthermore, it may also regulate other nuclear processes *via* the phosphorylation of additional nuclear substrates (see below).

## Signals and Post-Translational Modifications That Modulate the Cytoplasm to Nucleus Translocation of SRPKs

The “steady-state” cytoplasmic localization of SRPKs is altered by external signals that dissociate the kinases from the chaperone complexes, resulting in their translocation to the nucleus. Kinase activity is a prerequisite for the nuclear translocation of SRPKs. Blocking SRPK activity either by inactivating mutations or by the selective SRPK1/2 inhibitor, SRPIN340, efficiently impeded the entry of the kinases into the nucleus (Ding et al., 2006; Jang et al., 2009; Zhou et al., 2012; Koutroumani et al., 2017; Moreira et al., 2018; Sigala et al., 2021a).

Earlier reports indicated that cell cycle signals triggered the translocation of SRPKs to the nucleus at the late G2 phase in yeast and HeLa cells (Takeuchi and Yanagida, 1993; Ding et al., 2006) and suggested that phosphorylation was implicated in the observed relocation of the kinases (Takeuchi and Yanagida, 1993). Two protein bands corresponding to *Schizosaccharomyces pombe* dsk1 were observed in SDS-PAGE. The fast migrating form was enriched in interphase cells, while the slowly migrating form was prominent in mitotic cells. The disappearance of the slowly migrating band following phosphatase treatment strongly suggested that mitotic dsk1 was phosphorylated.

Activation of the PI3K-Akt signaling pathway by EGF was then shown to be involved in SRPK1/2 nuclear translocation. Specifically, Jang et al. (2009) reported that activated Akt phosphorylated SRPK2 on Thr492, thus promoting its nuclear import, while Zhou et al. (2012) reported that, even though SRPK1 was not a direct substrate of Akt, Akt induced the autophosphorylation of Thr326 and Ser587 located in the spacer and the C-terminal catalytic domain of SRPK1, respectively. This autophosphorylation event released the kinase from the chaperone complex and resulted in its relocation to the nucleus. By using specific inhibitors against components of the EGF pathway, Zhou et al. (2012) suggested that both SRPKs act below PI3K but above mTOR. Yet, some years later, Lee et al. (2017) showed that mTORC1 triggered a series of phosphorylation events leading to SRPK2 nuclear translocation. According to this cascade, mTORC1 phosphorylated and activated ribosomal S6 kinase 1 (S6K1), which in turn phosphorylated SRPK2 at Ser494, priming Ser497 phosphorylation by casein kinase 1 (CK1). Of note, Ser494 and Ser497 were also found phosphorylated by MS/MS spectrometric and Western blotting analysis in non-treated head and neck squamous cell carcinoma (HNSCC) cells (Radhakrishnan et al., 2016).

On the other hand, nuclear translocation of SRPKs is closely related to the DNA damage response. Vivarelli et al. (2013) reported that the nuclear accumulation of SRPK2 in neuroblastoma cells after oxidative and genotoxic stress was mediated by phosphorylation of Ser588 (Ser581 in mouse SRPK2). Even though this residue was considered by the authors to be a casein kinase 2 (CK2) site, on the basis of a previous mapping and analysis of SRPK1 phosphorylation sites (Mylonis and Giannakouros, 2003), and the fact that caffeine, a potent inhibitor of ATM/ATR kinases, blocked SRPK2 nuclear translocation strongly suggested that SRPK2 may act downstream of ATM/ATR. In this respect, we have recently shown that treatment of HeLa and T24 cells with 5-fluorouracil (5-FU) induced SRPK1 phosphorylation on two residues located within S/TQ motifs, Thr326 and Ser408, in an ATR/ATM-dependent manner, thus resulting in nuclear accumulation of the kinase (Sigala et al., 2021a). Furthermore, in cisplatin-treated cells, pharmacological inhibition of ATM or Chk2, which is the main kinase that functions as a downstream effector of ATM, prevented nuclear accumulation of SRPK2, whereas blocking the activity of ATR had only a marginal impact on the translocation of the kinase to the nucleus (Sigala et al., 2021b). These data suggest that nuclear SRPK2 may act downstream of Chk2 in the ATM/Chk2 cascade. Of note, cisplatin had little effect on the nuclear entry of SRPK1, implying that distinct signaling pathways are activated in response to these two chemotherapeutic agents.

In most of the above-mentioned phosphorylation events, phosphorylation was both necessary and sufficient to induce the relocation of the kinases from the cytoplasm to the nucleus, since the phosphorylation-defective mutants were restricted in the cytoplasm, whereas the phosphorylation-mimicking mutants localized in the nucleus even in the absence of any treatment. Yet, in 5-FU treated HeLa cells, while the phosphorylation-defective double mutant (SRPK1326/408A) was almost completely restricted to the cytoplasm, the phosphorylation-mimicking double mutant (SRPK1326/408D) partially localized in the nucleus in the absence of 5-FU treatment, suggesting that phosphorylation of these residues was necessary but not sufficient for nuclear translocation of SRPK1 and additional modification(s) was (were) required (Sigala et al., 2021a). In this respect, Edmond et al. (2011) showed that knockdown of Tip60 acetyltransferase in lung cancer cells resulted in strong nuclear accumulation of SRPK1 and SRPK2. Recently, Wang et al. (2020) identified by mass spectrometry analysis five lysine residues in SRPK1 that could be acetylated in a Tip60-dependent manner, Lys215, Lys258, Lys265, Lys301 and Lys318, while previously Choudhary et al. (2009) identified two additional lysine residues, Lys585 and Lys588. Mutation of all these seven sites to arginine induced nuclear translocation of SRPK1 in HeLa and MCF7 cells (Wang et al., 2020). There are no reports so far on which lysine residues of SRPK2 are acetylated by Tip60. Even though, blocking acetylation seemed to be sufficient to drive the nuclear import of SRPK1/2 and despite the observation that overexpressing a deacetylase did not have any impact on the subcellular localization of the kinases (Edmond et al., 2011), we favor a previously formulated hypothesis that an inverse correlation between SRPK1/2 acetylation and phosphorylation may modulate the kinase localization (Wang et al., 2020).

Finally, SRPK2 was also found to be O-GlcNAcylated at Ser490, Thr492, and Thr498 in non-treated MCF-7 and HEK293T cells (Tan et al., 2021). O-GlcNAcylation promoted interaction of SRPK2 with importin a, thus facilitating the nuclear translocation of the kinase. According to the authors, O-GlcNAcylation functions in parallel with the mTORC1/S6K1/CK1 pathway (Lee et al., 2017) and these two distinct types of post-translational modifications do not interfere with each other. Yet, blocking O-GlcNAcylation by O-GlcNAc transferase (OGT) knockdown or pharmacological inhibition abolished almost completely nuclear staining of SRPK2 (Tan et al., 2021).

**FIGURE 1:**
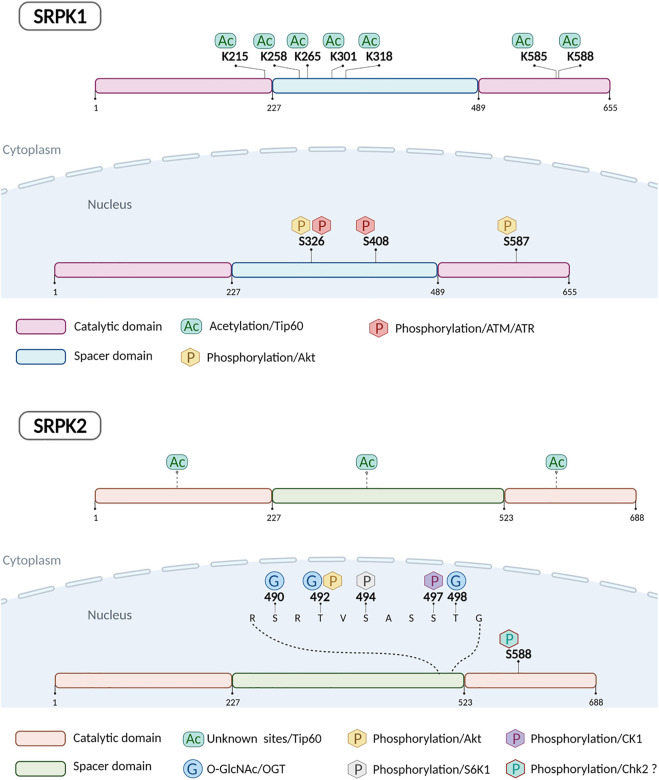
Schematic representation of human SRPK1 and SRPK2 indicating post-translationally modified amino acids and enzymes responsible.

Figure 1 illustrates the currently known post-translational modifications of SRPK1/2.

## Functional Significance of SRPK1/2 Nuclear Translocation

The balance between cytoplasmic and nuclear SRPK levels is critical for the cell. Shifting the balance in favor of nuclear localization should be tightly regulated by how and to what extent SRPKs are post-translationally modified in order to allow cells to respond appropriately to external signals. In this regard, activated Akt-induced autophosphorylation of SRPK1 resulted in partial relocation of the kinase to the nucleus in EGF-treated HEK293T cells (Zhou et al., 2012). Our *in vitro* assays revealed that activated Akt1 induced phosphorylation of SRPK1 at a very low stoichiometry (Sigala et al., 2021a). Yet, while these substoichiometric levels of phosphorylation are of low importance *in vitro*, they may be functionally significant in the cellular context, allowing a well-defined number of SRPK1 molecules to enter the nucleus. Partial nuclear translocation of SRPK1 was also observed in EGF-treated melanoma cells (Moreira et al., 2018). Both nuclear and cytoplasmic staining of SRPK2 were obtained in EGF-treated HEK293T cells or cells transfected with activated Akt, whereas an antibody against p-SRPK2 stained only the nuclei of certain cells, implying that a fraction of the kinase was phosphorylated and entered the nucleus (Jang et al., 2009 Supplementary data; Zhou et al., 2012). Furthermore, despite the vital role of O-GlcNAcylation in inducing nuclear translocation of SRPK2, thus promoting posttranscriptional *de novo* lipogenesis and cancer cell growth, in about 50% of MCF-7 cells no staining of SRPK2 was detected in the nucleus, while in the remaining 50%, SRPK2 was mainly cytoplasmic, suggesting that only a fraction of the kinase was O-GlcNAcylated and imported to the nucleus (Tan et al., 2021). Similarly, insulin induced partial nuclear translocation of SRPK2 that promoted splicing of lipid synthesis-related mRNAs, via activation of the mTORC1/S6K1/CK1 pathway (Lee et al., 2017).

On the other hand, genotoxic/stress signals largely induce nuclear accumulation of SRPKs, either by promoting the phosphorylation of the same or different sites with a significant increase in phosphorylation stoichiometry, and/or by the additive effect of a second modification such as deacetylation. Thus, nuclear SRPKs may also be regarded as key mediators of the cellular response to DNA damage (Edmond et al., 2011; Vivarelli et al., 2013; Sigala et al., 2021a; Sigala et al., 2021b).

**FIGURE 2:**
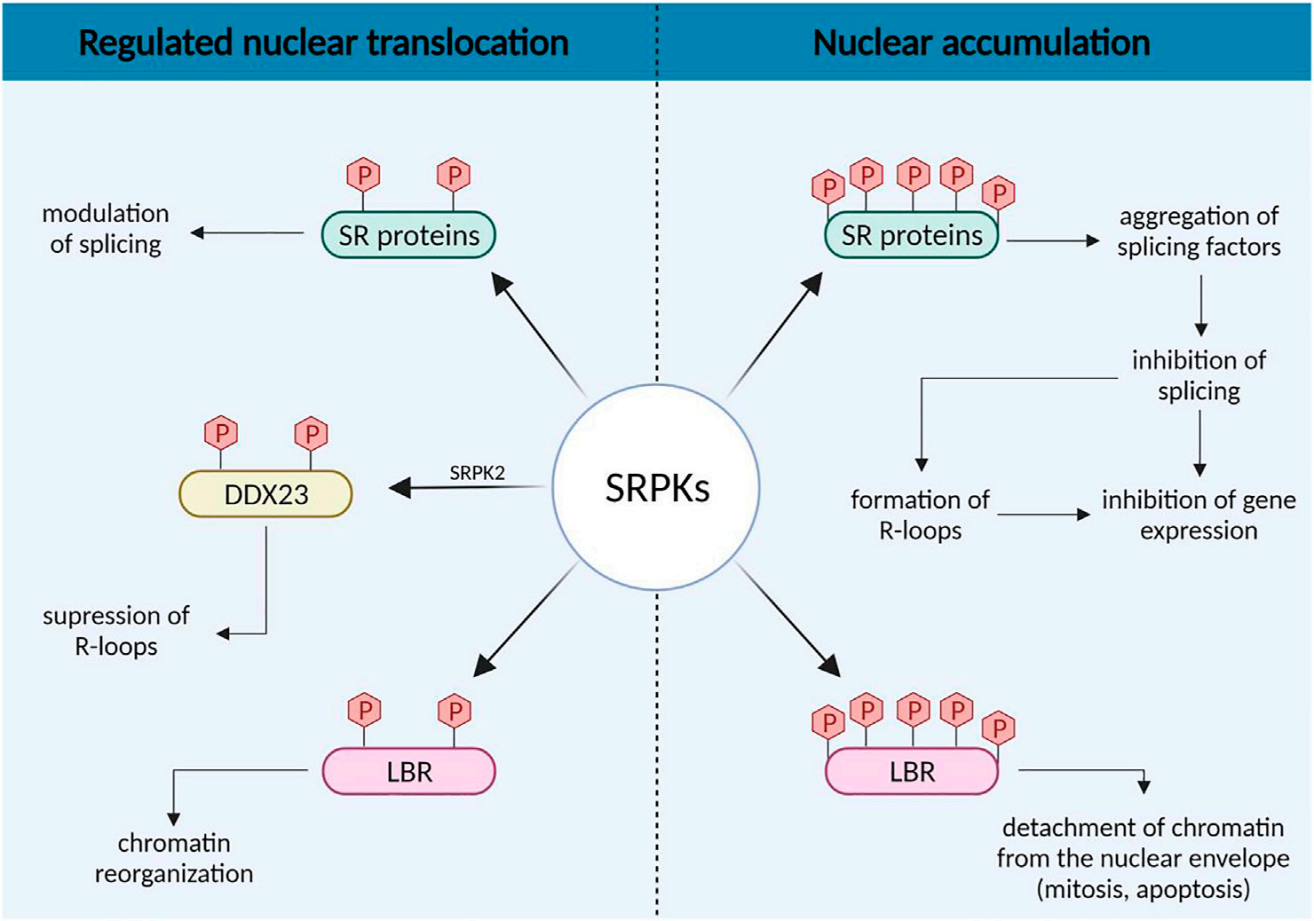
Concentration-dependent effects of SRPKs on splicing regulation and chromatin reorganization.

The increased nuclear levels of SRPKs were primarily, if not exclusively, associated with alterations of the splicing machinery (Figure 2). In general, it is believed that a growth factor/hormone-mediated increase in the nuclear concentration of SRPKs may alter the level of phosphorylation of SR proteins and consequently their activity on their primary transcript targets, favoring the expression of splicing isoforms that contribute to cell growth and promote tumorigenic properties (Zhou et al., 2012; Lee et al., 2017; Tan et al., 2021). On the contrary, nuclear accumulation of SRPKs, mediated by deletion of the spacer region, causes aggregation of splicing factors, which results in splicing inhibition and may lead to general inhibition of gene expression (Siebel et al., 1999; Ding et al., 2006).

The variations in SRPK nuclear levels may also have opposing effects on other nuclear activities of the kinases indirectly related to splicing, such as the formation of R-loops (Figure 2). R-loops are nucleic acid structures formed during transcription when the nascent RNA molecule hybridizes with the template DNA strand and may compromise genomic integrity (Sollier and Cimprich, 2015). Sridhara et al. (2017) reported that SRPK2 phosphorylates the DDX23 helicase, thus suppressing the formation of R-loops. However, RNA processing defects result in elevated levels of R-loops (Stirling et al., 2012; Montecucco and Biamonti, 2013). Hence, signals that promote the nuclear accumulation of SRPK2 and the subsequent dysregulation of the splicing machinery would also be anticipated to lead to the formation of R-loop structures. In this respect, it was also previously shown that R-loop formation confers sensitivity to cisplatin (Cai et al., 2020).

Additional nuclear events may be critically regulated by the nuclear levels of SRPKs, via the phosphorylation of proteins other than SR splicing factors (Figure 2). Lamin B Receptor (LBR), a key factor tethering peripheral heterochromatin (Solovei et al., 2013), is a well-characterized substrate of SRPK1 and possibly of SRPK2 (Nikolakaki et al., 1996; Sellis et al., 2012). Phosphorylation plays a significant role in regulating the attachment of LBR to chromatin (Takano et al., 2004). Thus, a regulated increase in the nuclear levels of SRPKs, mediated by an external signal, may fine-tune gene expression through the detachment of specific chromatin regions from the nuclear periphery and their subsequent displacement to a transcriptionally active microenvironment (for reviews see Misteli, 2005; Nikolakaki et al., 2017). On the other hand, nuclear accumulation of SRPKs mediated by cell cycle or genotoxic/stress signals may lead to complete detachment of chromatin from the nuclear envelope. Such a detachment is indeed observed *in mitotic* prophase and this contributes to *nuclear envelope* breakdown (Guttinger et al., 2009) and also at early stages of the apoptotic process prior to nuclear envelope breakdown, chromatin condensation and nuclear fragmentation (Lindenboim et al., 2020).

A direct role for SRPK1 in the transcription-related DNA damage response has also been proposed by Boeing et al. (2016). In this study a genome-wide siRNA screen assessing gene products that affect transcription after UV-irradiation was performed, while in parallel the UV-induced phosphoproteome was analyzed. SRPK1 was one of the main kinases that scored in the RNAi screen and was also associated with 20 proteins showing UV-induced phosphorylation. The three most highly phosphorylated SRPK1-interacting proteins were the tumor-associated genes BCLAF1 and THRAP3, and apoptotic chromatin condensation inducer 1 (ACIN1), all three of which were found to interact with RNA Polymerase II. Furthermore, SRPK1, BCLAF1, THRAP3, and ACIN1 were associated with several networked proteins that also scored in the RNAi screen, suggesting that SRPK1 is part of a network involved in the transcription-related DNA damage response.

## Opposing Effects of SRPK Knockdown/Inhibition

**FIGURE 3:**
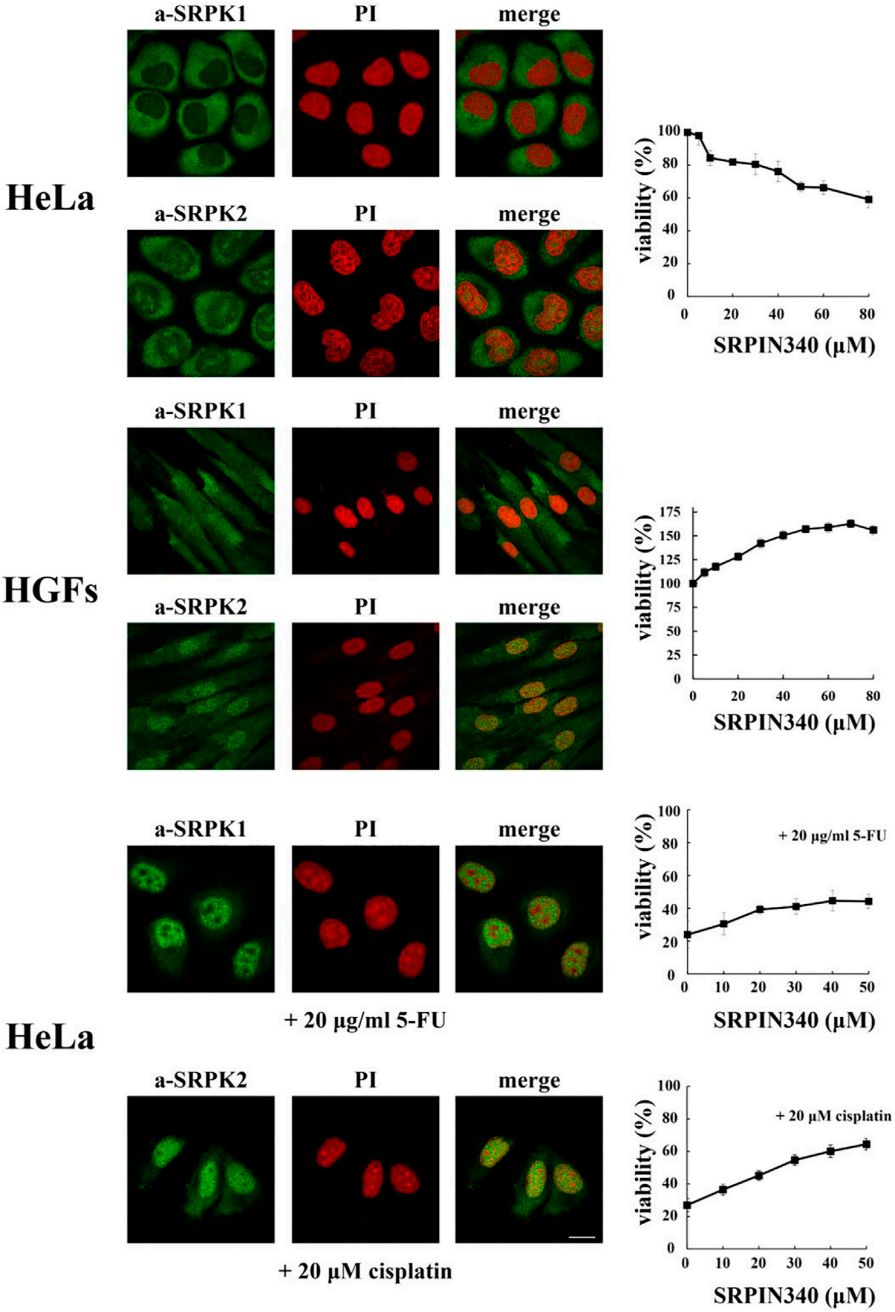
Fluorescent patterns of SRPK1 and SRPK2 in HeLa cells, human gingival fibroblasts (HGFs), HeLa cells treated with 20 μg/ml 5-FU for 48 h and HeLa cells treated with 20 µM cisplatin for 24 h (left panels). SRPK1 and SRPK2 were detected using the respective anti-SRPK1 and anti-SRPK2 monoclonal antibodies, while the nuclei were stained with PI. Scale bar: 10 µm. In each case the effect of kinase inhibition, by SRPIN340, on the number of viable cells was measured using an MTT assay (right panels). Viability is expressed as a percentage of the viability of untreated cells, which was set to 100 percent. The data on HeLa cells were taken from Sigala et al. (2021a) and Sigala et al. (2021b).

SRPKs are critical in promoting cancer cell proliferation and viability. There are numerous reports showing that down-regulation or pharmacological inhibition of the kinases results in decreased proliferative capacity and increased apoptotic potential (Figure 3; see also Nikas et al., 2019; Naro et al., 2021 and references therein). However, findings from normal cells or near normal cells, i.e., immortalized but non-transformed cells, question the notion that the predominant cytoplasmic localization of SRPKs is associated with cell proliferation. Specifically, vascular smooth muscle cells treated with siRNA-SRPK1 exhibited enhanced cell proliferation, repressed cell apoptosis, and increased vascular remodeling (Li and Wang, 2019). Furthermore, knocking down SRPK1 in immortalized MEFs lacking p53 (p53−/−) promoted cell transformation resulting in significant anchorage independent growth. These immortalized SRPK1-null MEFs were also able to develop into tumors when injected into nude mice, while on the contrary wild type MEFs immortalized by the T antigen were not tumorigenic (Wang et al., 2014). Since the subcellular localization of SRPK1 was not examined in these reports, we performed an initial immunofluorescent study in human gingival fibroblasts which are the main cellular constituent of gingival tissue and are embryo-like cells with the capacity of self-renewal. As shown in Figure 3 and in accordance with previously reported immunohistochemical data (Mytilinaios et al., 2012), SRPK1 localized mainly in the cytoplasm but a nuclear staining was also clearly observed, while SRPK2 showed more marked nuclear localization. Interestingly, blocking SRPK1/2 activity by SRPIN340 in these cells promoted cell proliferation (Figure 3).

On the other hand, nuclear accumulation of the kinases mediated by genotoxic signals was shown to be actively involved in drug toxicity and thus knocking down or inhibiting the activity or preventing the nuclear entry of the kinases neutralized the effects of the drug. Specifically, blocking SRPK1 activity by SRPIN340 or blocking the activity of the ATR/ATM cascade by pharmacological agents prevented the nuclear accumulation of SRPK1 in 5-fluorouracil (5-FU)-treated HeLa cells and conferred partial resistance to the drug (Figure 3; see also Sigala et al., 2021a). Similarly, blocking SRPK2 nuclear accumulation in cisplatin-treated HeLa cells neutralized the effect of cisplatin (Figure 3; see also Sigala et al., 2021b), while inhibition of SRPK1/2 by SRPIN340 protected cardiomyocytes from oxidative stress-induced apoptosis and cell death (Huang et al., 2019). Furthermore, in cisplatin treated non-small cell lung cancer cells Tip60 acetyltransferase was down-regulated resulting in nuclear accumulation of SRPK2 and cell apoptosis (Edmond et al., 2011). Apoptosis was impaired in kinase-depleted cells, highlighting the crucial role of nuclear SRPK2 in the apoptotic process. Interestingly, high levels of Tip60 have been associated with cisplatin resistance in prostate, epidermoid and lung cancer cells (Miyamoto et al., 2008).

There has been considerable debate within the literature as to whether SRPKs are related to chemotherapy sensitivity or resistance. The conflicting results were derived mainly from studies on SRPK1 in cisplatin-treated cells (Schenk et al., 2001; Schenk et al., 2004; Hayes et al., 2006; Plasencia et al., 2006; Hayes et al., 2007; Krishnakumar et al., 2008; Odunsi et al., 2012). In all these reports only the protein levels of SRPK1 were correlated to drug responsiveness. While nuclear accumulation of SRPK1/2 mediates drug toxicity, cisplatin induces rather limited nuclear translocation of SRPK1, the extent of which may vary between different cell types (Schenk et al., 2001; Edmond et al., 2011; Sigala et al., 2021a). The different nuclear levels of the kinase may account at least partially for the observed divergent data. Yet, apart from the subcellular localization, the sensitivity of a cancer cell line to cisplatin seems also to be critical in determining the role of SRPK1 in drug responsiveness. In this regard, the most highlighted example, where SRPK1 expression was associated with both cisplatin sensitivity and resistance, is ovarian cancer. Schenk et al. (2001) used the A2780 cell line which represents the most common histology type of ovarian cancer and is very sensitive to cisplatin, while Odunsi et al. (2012) used the SKOV3 cell line which exhibits about ten times lower sensitivity to cisplatin than the A2780 cell line. Actually, SKOV3 cells were even more resistant to cisplatin than A2780 resistant cells (Odunsi et al., 2012). Reduction of SRPK1 expression using shRNAs in SKOV3 cells led to enhanced sensitivity to cisplatin (Odunsi et al., 2012), whereas down-regulation of SRPK1 by antisense treatment induced resistance of A2780 cells to cisplatin (Schenk et al., 2001). Notably, there seems to be a threshold concentration of the drug, above which, the cytotoxic effects cannot be reversed (Schenk et al., 2001; Sigala et al., 2021a). In accordance with their ovarian cancer study, Schenk et al. (2004) reported later that cisplatin responsiveness of male germ cell tumors, which are among the most cisplatin-sensitive tumors, closely correlated with high levels of SRPK1 expression, while a similar observation was also made by Krishnakumar et al. (2008) in archival Rb tumors. On the other hand, in breast (MCF7 and MCF7A), colon (Caco2 and HT29) and pancreatic (MiaPaCa2 and Panc1) cancer cell lines that were either insensitive (MCF7, MCF7A, Caco2, HT29) or exhibited low sensitivity to cisplatin (MiaPaCa2, Panc1), under the treatment conditions used by the authors (10 μM cisplatin for 24 h), down-regulation of SRPK1 by siRNA encoding constructs potentiated the effect of cisplatin (Hayes et al., 2006; Hayes et al., 2007). These data are in line with a proposed exclusive cytoplasmic localization of SRPK1 in cisplatin-treated breast cancer cells due to an unanticipated increase of Tip60 levels in these cells (Wang et al., 2020), which is contrary to the data presented by Miyamoto et al. (2008) and Edmond et al. (2011). Moreover, cisplatin-resistant MDA-MB-231 and MCF7 cells could also be re-sensitized by inhibiting SRPK1 activity using SRPIN340 (Wang et al., 2020).

The observation that aberrant nuclear accumulation of SRPK2 has toxic effects on cancer cell proliferation and survival seems also to be overturned by the findings on LAM 621-101 cells that carry inactivating mutations in both alleles of the Tuberous Sclerosis Complex 2 (TSC2) gene (TSC2^−/−^ cells). TSC2 is a negative regulator of mTORC1 and therefore in these cells the mTORC1/S6K1/CK1 pathway is constitutively activated, SRPK2 is highly phosphorylated and as expected was found almost exclusively to the nucleus (Lee et al., 2017). Primary LAM 621–101 cells stop proliferating and enter into senescence after a few passages due to p53 amplification (Zhang et al., 2003; Yu et al., 2004). To circumvent premature senescence the cells have to be immortalized. LAM 621-101 cells used by Lee et al. (2017) were immortalized by expression of the HPV16 E6 and E7 genes as well as human telomerase (Siroky et al., 2012). The immortalized cells showed high rates of proliferation and were highly tumorigenic. This artificial route to boosting cell proliferation and growth may reprogram the cells to take advantage of the high levels of SRPK2 nuclear activity in order to meet their intense lipid requirements. Accordingly, supplementation with fatty acids and cholesterol partially restored the growth of cells in which SRPK2 was knocked-down or inhibited by SRPIN340. Interestingly, Lee et al. (2007) proposed that while mTOR inhibitors may exert protective effects against DNA damage agents to benign tumors such as tuberous sclerosis, they enhance the effects of chemotherapeutics in tumors such as non-small-cell lung carcinomas and ovarian cancer.

## Conclusion

Based on our recent data (Sigala et al., 2021a; Sigala et al., 2021b) we formulated the hypothesis that the predominant cytoplasmic localization of SRPKs is associated with cell proliferation, whereas nuclear accumulation of the kinases is closely related to inhibition of growth. The best evidence in support of this hypothesis comes from experiments showing that forced nuclear accumulation of SRPKs due to deletion of their spacer region, without any other cell treatment, results in harmful effects. Yet, the cell state seems also to have an important role in determining SRPK function. In cells showing a high proliferation rate (cancer cells, immortalized highly tumorigenic cells, cells resistant to drugs) SRPKs are critical in maintaining the high proliferation rate and thus, they may exert a tumor promoting function, whereas in cells with a lower proliferation rate (normal mammalian cells, immortalized but not tumorigenic cells) SRPKs are critical in maintaining the lower proliferation rate and the non-tumorigenic features and thus they may exert a tumor suppressor function. Furthermore, in non-proliferating cells (primary TSC2^−/−^ cells, sensitive cancer cells treated with chemotherapeutic drugs) nuclear SRPKs are critical in sustaining the non-proliferative state and/or mediating drug toxicity. This suggests that SRPKs have a complicated role in cancer that seems to depend on the cell state, the subcellular localization and the threshold levels of the kinases. Deciphering this coordinated mode of action not only can provide valuable insights into the roles of SRPKs but can have a significant impact on medical research.
